# Characterization of agar extracted from *Gracilaria* species collected along Tanzanian coast

**DOI:** 10.1016/j.heliyon.2022.e09002

**Published:** 2022-02-22

**Authors:** Said A.H. Vuai

**Affiliations:** Department of Chemistry, College of Natural and Mathematical Sciences, The University of Dodoma, United Republic of Tanzania

**Keywords:** Agar, Gel strength, *G*. *salicornia*, *G*. e*dulis*, *G*. c*orticata*, Alkali treatment, Seaweed

## Abstract

The study was conducted to investigate the effect of spatial variation, alkali treatment and the volume of extractant on yield and gel strength of agar for three *Gracilaria* species (*G*. s*alicornia*, *G*. e*dulis* and *G*. c*orticata*) collected from the Tanzanian coast (Dar es Salaam, Tanga and Zanzibar). Treated and untreated *G.* c*orticata* showed the highest yield (27 ± 0.7 % and 26.2 ± 1.3 % for treated and untreated, respectively), followed by *G. salicornia* then *G. edulis*. *G. salicornia* collected from Zanzibar showed the highest mass yield (22.9 ± 4.3 % for treated) followed by those collected from Tanga. Varying the volume for extraction showed no significant difference in mass yield where the p-value was >0.05. The highest gel strength was recorded from treated *G. salicornia* collected from Tanga (495 ± 29.5 gcm^−2^). Gel strength varied significantly between species. Spatial variability showed a significant difference in gel strength; the sample collected from Tanga showed the highest gel strength, followed by Zanzibar then Dar es Salaam. The variation due to the volume of distilled water used for extraction showed no significant difference in gel strength at a p-value >0.05. The highest gel strength was recorded at the volume of 1500 mL (467.5 ± 98.4 gcm^−2^), and the smallest gel strength was recorded at 500 mL. In all cases, there was a significant difference in mass yield and gel strength between treated and untreated samples. *G. salicornia* showed promising results as a local source of agar as it showed the highest gel strength though it produced an intermediate amount of agar. Based on the finding of this study, the volume of extraction of agar should be maintained as 1000 mL because by increasing the volume of extraction from 1000 mL to 1500 mL, the agar yield and gel strength don't change significantly. Agar yield and gel strength of *Gracilaria* species (*G. salicornia, G. edulis and G. corticata*) can be improved by alkali treatment, but further study is needed to determine the optimum amount and concentration of alkali to be used that will produce maximum yield and gel strength.

## 1 Introduction

Currently, the demand for agar in Africa, including Tanzania, is growing rapidly due to the increasing number of biotechnology and microbiology laboratories. However, the supply system depends wholly on importation. In Tanzania, the problem remains big despite being a home of several species of seaweeds, which are the major raw material for agar production.

The genus Gracilaria is of considerable economic importance as an agarophyte, and it is the most abundant and promising resource of agar production. It has more than 150 species, distributed mainly in the temperate and subtropical zones (Radiah et al., 2011). The value of *Gracilaria* has increased with demand because of the high production cost of the agar from the genus *Gelidium* and insufficient wild stock of this genus. Therefore, more than half of the world agarophyte tonnage consists of *Gracilaria* (McHugh, 1991). Different species of *Gracilaria* include *G. edulis, G. corticata, G. millardetii, G. debilis* (formally *G. fergusonii*) *and G. salicornia* have been reported to be potential sources of agar along the Indian Ocean water, including Tanzania (Jaasund, 1976; Kappanna and Rao 1963; Guiry and Guiry, 2021).

The yield and physical properties of agar such as gel strength, gelling and melting temperature define its value to industries. The gel-forming properties of agar are widely used in the pharmaceutical, cosmetics, and food industries (Marinho-Soriano, 2001). The structure of agar consists of alternating β-1,3 and α-1,4 linked D and L galactose residues, respectively. The charged residues are present on the polysaccharide chain, of which the most frequent substituents are sulfate esters and pyruvate ketal groups (Lahaye and Rochas 1991). The gel properties are highly dependent on the amount and position of sulfate groups and the amount of 3,6-anhydrogalactose fraction of the phycocolloid (Duckworth and Yaphe, 1971). However, *Gracilaria* spp. contain several structures with different substitutes such as sulfate esters, methoxyl and pyruvic acids (Andriamanantoanina et al., 2007). The amount of these substituents affects the physical properties of the gel. Craigie (1990) stated that the molecular structure of agar polysaccharides from the genus *Gracilaria* appears as species-specific, particularly the type and location of sulfate esters.

Species is not the only factor of variance in yield and quality of agar but factors such as environmental conditions and seasonal variations (Bird, 1988), physiological factors (Craigie et al., 1984) and extraction methods (Freile-Pelegrin and Robledo, 1997) affects the relative proportion of algal constituents. Generally, *Gracilaria* species produce agars with low quality due to high sulfate content, and therefore, they are called agaroides. However, transformations of agaroides into real agar can be done by alkali treatment, which converts L-galactose- 6-sulfate to 3,6-anhydro-L-galactose by the treatment of *Gracilaria* with sodium hydroxide (Armisen, 1995).

The present research aimed to study the influence of spatial variation, alkali treatment and the volume of extractant on the characteristics of agar (Mass yield and gel strength) produced from three Gracilaria species *G. edulis, G. corticata and G. salicornia* collected along the coast of Tanzania.

## 2 Material and methods

### 2.1 Sample collection and preparation

**Figure 1:**
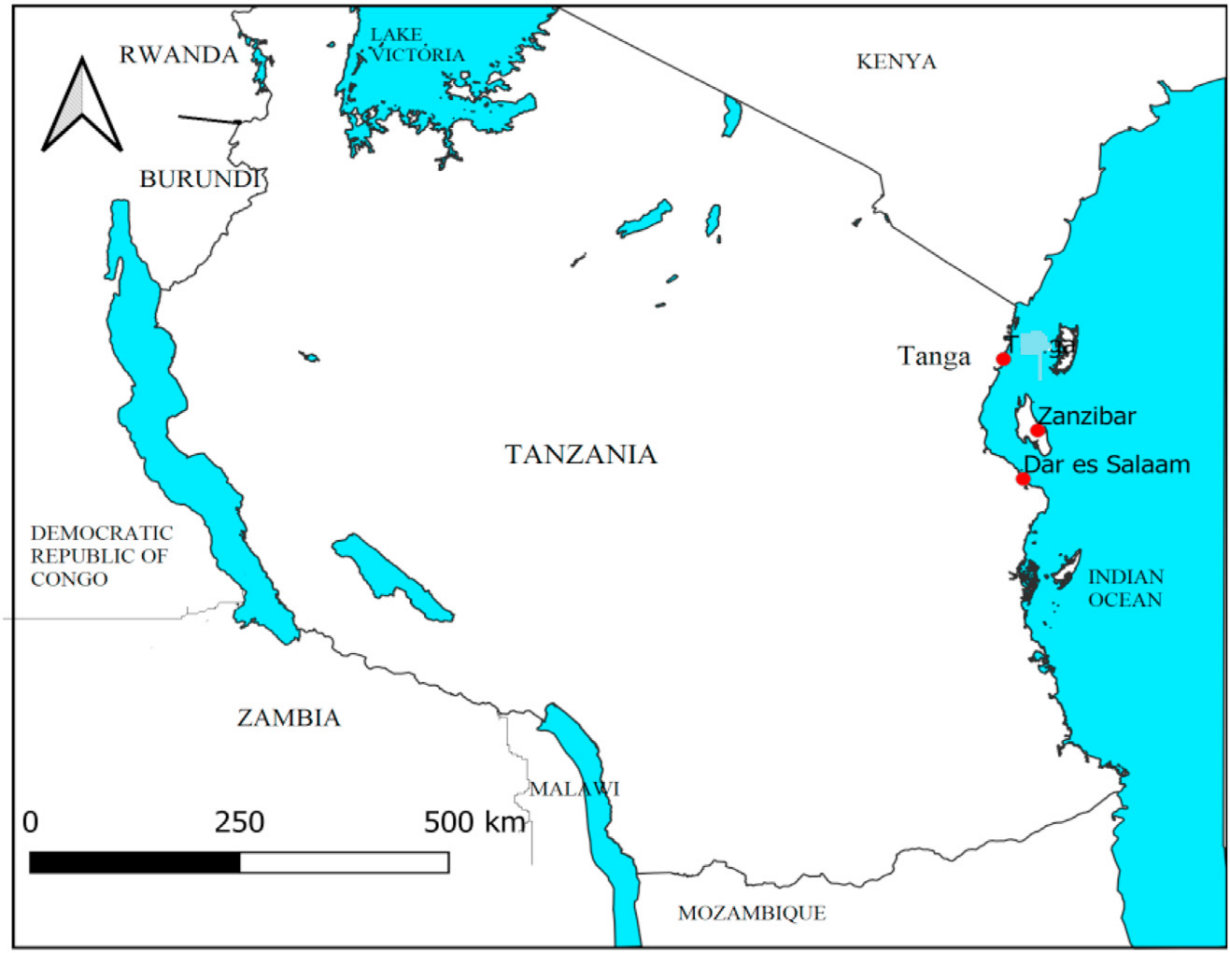
Map of Tanzania showing sampling location.

Specimens of *G*. *salicornia*, *G*. *edulis* and *G*. *corticata* were collected from natural stocks from three different locations along the coast of Tanzania (Zanzibar, Tanga and Dar es Salaam) as shown in Figure 1. The collected samples were washed using seawater to remove sands and other contaminants then washed thoroughly with tap water and exposed to sunlight for bleaching. The bleaching procedures were repeated for two weeks. Finally, the bleached samples were stored in plastic bags until the time for agar extraction.

### 2.2 Alkali treatment

Bleached samples were soaked in 350 mL of 20 % aqueous NaOH for three days at room temperature, then washed with water until the pH ranged between 7-8. The samples were then sun dried for 2 h before being mixed with 1000 mL of distilled water. The pH of the mixture was adjusted to 5.6 by adding acetic acid and allowed to dry for 30 min.

### 2.3 Agar extraction

A portion of 25 g of bleached samples of *G. salicornia, G. corticata* and *G. edulis* from three different sites (Tanga, Zanzibar and Dar es Salaam) were used for agar extraction with and without alkali treatment. Different portions of samples (25 g) were mixed with 1000 mL of distilled water and then placed in a water bath at 95 °C for 1 h. Another set of experiments was conducted by extracting 25 g of samples using three different volumes (1500 mL, 1000 mL and 500 mL) of distilled water. The resulted samples were homogenized in a blender and immediately put in a filter cloth (nylon) with the addition of 100 mL of hot distilled water and squeezed to allow the filtration to take place. The agar solution obtained was poured in an aluminium pan. The solution was cooled and allowed to gel at room temperature. The gel was cut into square bars and then frozen for 24 h in a deep freezer. The frozen gel was thawed, washed with distilled water, and dried in the sun for 1 day and then in an oven at 60 °C to a constant weight. Agar was then weighed and ground in a Micro Hammer Mill "Culatti" to a 100-mesh powder. The percentage yield of agar was calculated using the formula below:

Agar Yield (%) = [(Dry weight of agar (g)/ Dry weight of seaweed (g))] × 100

Gel strength (gcm^−2^) was measured by taking a portion of 50 mL of 1.5 % w/v solution of extracted and standard agars, each one separately and autoclaved at 120 °C for 30 min. After the gel formation at room temperature, the gel was stabilized at 5 °C for overnight in a refrigerator. The gel strength of agar was then measured at 20 °C by Brookfield CT3 Texture Analyzer (Brookfield Engineering Labs., Inc) using a cylindrical probe (TA10 Cylinder 12.7 mm diameter, 35 mm long). Sulfate was determined using the infrared (IR) spectrophotometry method using a Perkin–Elmer 983G. The relative absorbance ratio was calculated at 930/2920 and 1250/2920 cm^−1^ for 3,6-anhydrogalactose and total sulfates, respectively (Lahaye and Yaphe, 1988). All data were analyzed using Statistica 7 and Origin Pro 8.5.

### 2.4 FTIR spectroscopy of agar

Two grams powder of extracted agar was mixed with potassium bromide to prepare a solid disc and FTIR spectra were collected from 4000-500 cm^−1^ range in transmission mode with 2 cm^−1^ resolution over 10 scans by using spectrometer.

## 3 Results and discussion

### 3.1 Variation of mass yield and Gel strength between *Gracilaria* species

**Figure 2:**
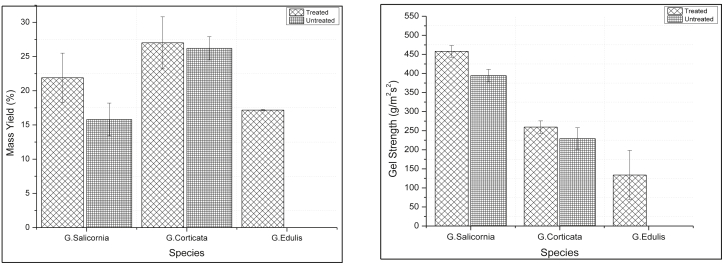
Percentage mass yield (Left) and gel strength (Right) of Gracilaria species.

Table 1 summarizes the results of Two-Way ANOVA. The agar yield and gel strength from the three species varied significantly, where p-value was >0.05. For both treated and untreated experiments *G. corticata* specie showed highest mass yield 27 ± 0.7 % (treated) and 26.2 ± 1.3 % (untreated) followed by *G. salicornia* 21.9 ± 0.7 % (treated) and 15.8 ± 0.7 % (untreated), while *G. edulis* specie show smallest mass yield 17.2 ± 1.6 % (treated) as shown in Figure 2. It is well established that, agar yield of *Gracilaria* species varied among each other (Arminsen, 1995). The agar content of *G*. e*dulis* specie recorded in this study differs from that observed from the study conducted by Ganesan and Subba Rao (2004). The agar content was higher in all conditions compared to what was observed in this study. They found that the agar content ranged from 18.5 to 50.3 % for untreated samples, acid-treated agar content ranged from 30.5 to 60.8 %, the agar content for 1 % KOH ranged from 30 to 60.6 % and for 0.5 % of acetic acid + 1 % of KOH agar content ranged from 55.3 to 69.2 %. The agar content for *G.* c*orticata* species recorded in this study was higher compared to that recorded by Ramavatar et al., (2008) where they obtained values ranging from 16 ± 0.8 to 9.5 ± 0.1 %. These differences may be due to the difference in geographical location and extraction methods used, as reported by Kumar and Fotedar (2009). The agar yield for *G. salicornia* recorded in this study is closely similar to that recorded by Buriyo and Kivaisi in 2003 where the agar yield ranged from 13.7 to 30.2 %. The possible reasons for these similarities can be the similar geographical location of samples.

**Table 1:** Summary of Variation of Mass yield and Gel strength among *Gracilaria* species.

Effects	df	Mass Yield (%)		F	P	Gel strength (gm^−2^s^−2^)		F	P
		MS	SS			MS	SS		
Species	2	412.8	825.5	37.1	<0.001	328175.83	656351.66	64.139	<0.001
Treatment	1	272.5	272.5	24.5	<0.001	62629.4	62629.4	14.19	<0.001
Error	62	11.1	689.6			4413	269223.5		
Total	65		1936.7				983775.8		
Spatial	2	39.3	78.6	5.0	0.012	20886.007	41772.014	10.126	<0.001
Treatment	1	387.7	387.7	49.2	<0.001	81700.694	81700.694	19.725	<0.001
Error	34	7.9	267.9			2062.571	70127.431		
Total	37		703.5				192181.579		
Volume	**2**	**31.9**	**63.9**	**3.2**	**0.062**	**4929.1667**	**6858.33**	**1.427**	**0.26**
Treatments	1	221.2	221.2	22.2	<0.001	60501.042	60501.042	17.520	<0.001
Error	20	10.0	191.6			3453.229	69064.583		
Total	23		484.3				139424.958		

MS = Mean Squares, SS = Sum of Squares, df = Degree of Freedom.

Bolded value show no significant difference.

*G. salicornia* specie show highest gel strength of 458 ± 15.5 gcm^−2^ (treated) and 394.4 ± 16.4 gcm^−2^ (untreated) followed by *G. corticata* 259.4 ± 16.4 gcm^−2^ (treated) and 229.2 ± 28.3 gcm^−2^ (untreated) while smallest gel strength was recorded from *G. edulis* specie 133.8 ± 64.4 gm^−2^s^−2^ (treated). The gel strength of *G. edulis* observed in this study is higher compare to that recorded by Ganesan and Subba Rao (2004), where they have recorded a maximum of 96.5 ± 2.9 gcm^−2^. Ramavatar et al., (2008) record a maximum of 490 ± 8.3 gcm^−2^ gel strength for *G. edulis* specie and 110 ± 8.9 gcm^−2^ for *G. corticata* species, which differs from the finding of the present study. These differences can be due to the difference in extraction processes, length of time of treatment and concentration of alkali.

### 3.2 Effect of spatial variation on mass yield and gel strength

**Figure 3:**
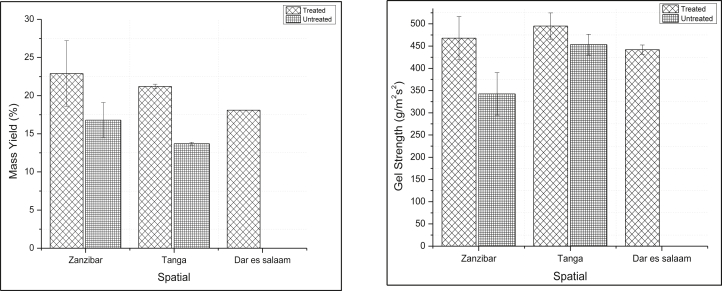
Spatial variation mass yield (Left) and gel strength (Right) of *Gracilaria* s*alicornia* samples in three study sites.

Spatial variation showed a significant difference in mass yield and gel strength, where p-value >0.05. *G.* s*alicornia* collected from Zanzibar showed highest mass yield 22.9 ± 4.3 % (treated), 16.8 ± 2.3 % (untreated) followed by those collected from Tanga 21.2 ± 0.3 % (treated), 13.7 ± 0.2 % (untreated) and Dar es salaam showed the smallest mass yield of 18.1 % (treated). Samples collected from Tanga showed the highest gel strength 495 ± 29.5 gcm^−2^ (treated), 453.3 ± 23.4 gcm^−2^ (untreated). In contrast, a sample from Dar es salaam showed smaller gel strength (442 ± 10.6 gcm^−2^) for treated compared to those which were collected from Zanzibar 467.7 ± 48.8 gcm^−2^ (treated), 342.5 ± 47.6 gcm^−2^ (untreated) as it shown in Figure 3. Buriyo and Kivaisi (2003) recorded the highest gel strength of 270 gcm^−2^ which is smaller than that recorded in this study. Different studies showed that, seaweed collected from different geographical locations differ in agar yield and gel strength. This is because they experience different environmental parameters like nutrients, which can affect seaweed's mass yield and gel strength. Buriyo and Kivaisi (2003) recorded different mass yield and gel strength between Dar es Salaam and Chwaka (Zanzibar), in contrast to the present study, they have recorded higher mass yield in Dar es salaam then in Zanzibar, but gel strength was higher in Zanzibar then Dar es Salaam similar to the present study.

### 3.3 Effect of volume of extraction on mass yield and gel strength

**Figure 4:**
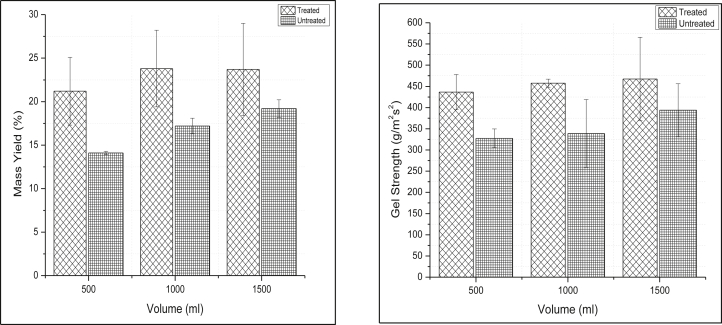
Effect of volume of extractant on mass yield (Left) and gel strength (Right) of Gracilaria species.

Difference in the volume of extraction used showed no significant difference in mass yield and gel strength where the p-value is > 0.05 (Figure 4). For the treated experiment, the highest mass yield was recorded at both 1500 mL and 1000 mL where the yield was 23.7 ± 5.3 %, and 23.4 %, respectively and the smallest mass yield was recorded at 500 mL 21.2 ± 3.9 %. For untreated experiment, the yield was 19.2 ± 1 %, 17.2 ± 0.9 % and 14.1 ± 0.2 % for 1500 mL, 1000 mL and 500 mL respectively. The highest gel strength was recorded at 1500 mL 467.5 ± 98.4 gcm^−2^ (treated) and 393.8 ± 62.4 gcm^−2^ (untreated) and the smallest gel strength recorded at 500mL 436.3 ± 41.5 gcm^−2^ (treated) and 327.5 ± 22.2 gcm^−2^ (untreated).

### 3.4 Effect of alkali treatment

Generally, there was a significant difference in mass yield between treated and untreated samples in all species, sites, and varying the volume of distilled water as shown in Table 1. In this study mass yield and gel strength were higher for alkali-treated then untreated samples. Based on the finding of this study, the highest mass yield observed was 27 ± 0.7 % (treated) for *Gracilaria,* which appears to be higher compared to that reported by Radiah et al., (2011) where the mass yield of both alkali-treated and untreated ranged from 7.1 % to 13.5 %. Radiah et al., (2011) also find that alkali-treated show low mass yield compared to untreated samples, which differ from the present study. This difference may be due to the difference in concentrations of alkali (NaOH) used, and the length of time of treatment. However, according to Ganesan and Subba Rao (2004) pre-extraction treatment using alkali or acid was observed to facilitate the retention of higher yield compared to untreated samples.

### 3.5 FT-IR spectra analysis of agar sample

The results of FT-Infra red spectra of agar samples were compared with that of standared Difco agar and the results reported by Balkan et al. (2005) as indicated below:

**Sample agar:** 650, 720, 780, 850, 1030, 1120, 1250, 1350, 1420, 1480, 1580, 1620, 2300, 2880, 3580, 3800 cm^−1^**.**

**Difco agar**: 650, 690, 713, 771, 869, 891, 931, 989, 1045, 1072, 1218, 1251, 1544, 1643, 2933 cm^− 1^ (Balkan et al., 2005).

**Figure 5:**
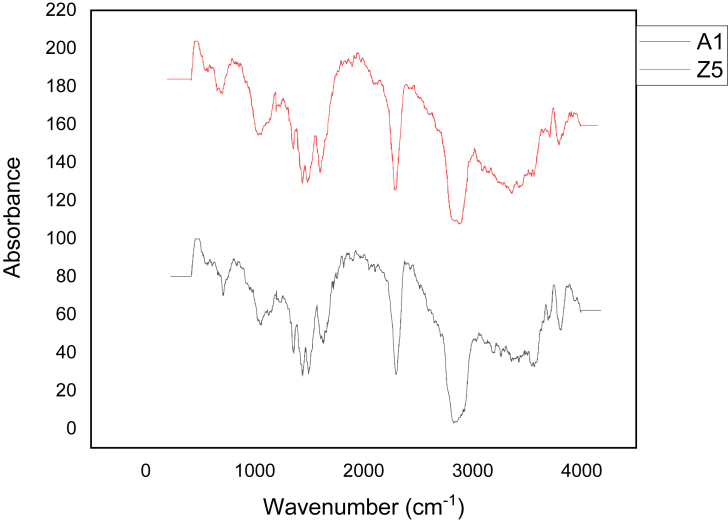
FT-IR spectrum of A1- Standard Difco-agar and Z5 *Gracilaria salicornia* agar sample.

The spectrum of agar from *Gracilaria salicornia* shows similarities with those of Standard Difco agar (Figure 5). Both show the characteristic bands at 650, 1030 and 1250 cm^−1^. Other bands which were found in *G. salicornia* with approximately the same values as in Difco agar were 720 (713), 780 (771, 778), 1350 (1362), 1420 (1434) cm^−1^. On the other hand, the bands at 850, 1120, 1480, 1620, 2300, 2880, 3580, 3800 cm^−1^ appear only in *G. salicornia* agar sample.

The absorbance between 720 -770 cm^−1^ indicates the existence of skeletal bending of the galactose ring. The band at 850 cm^−1^ also indicates the presence of D-galactose-4-sulphate. The absorbance at 1250 and 850 cm^−1^ indicates the presence of sulphate esters. This result is similar to earlier observations (Cabassi et al., 1978). The absence of IR bands at 705, 805 and 1070 cm^−1^ indicates low sulphate groups in extracted agar. The bands at 1420 and 1350 cm^−1^ are common to all spectra of polysaccharides associated with stretching of CH_3_**/**CH_2_ groups. The band at 890 cm^−1^ was found in Difco agar and absent in G. *salicornia* agar sample. This band is attributed to anomeric C–H of beta galactose residues. The absorbance at 930 and 1070 cm^−1^ found in Difco agar and absence in the sample agar is attributed to 3,6 anhydrogalactose. The absorbance at 1030 cm^−1^ present in sample agar and Difco agar, is assigned to C–C and C–O stretching vibrations of pyranose ring common to all polysaccharides. It was concluded that the analysis of sample IR-spectra confirm the extracted polysaccharides is an agar.

## 4 Conclusion

The amount of agar content (yield) and gel strength varied between species, sites, and extraction volume. *G.* s*alicornia* showed some promising results as a local source of agar as it showed the highest gel strength though it produced an intermediate amount of agar. Based on the finding of this study, the volume of extraction of agar should be maintained as 1000 mL because increasing the volume of extraction from 1000 mL to 1500 mL doesn't change the agar yield and gel strength significantly. Agar yield and gel strength of *Gracilaria* species can be improved by alkali treatment but further study is needed to determine the optimum amount and concentration of alkali to be used to produce maximum yield and gel strength at a minimum cost. Based on the analysis of sample IR-spectra, the extracted polysaccharides is suggested to an agar.

## Declarations

### Author contribution statement

Said Vuai: Conceived and designed the experiments; Performed the experiments; Analyzed and interpreted the data; Wrote the paper.

### Funding statement

This work was supported by 10.13039/501100005883Tanzania Commission for Science and Technology.

### Data availability statement

Data will be made available on request.

### Declaration of interests statement

The authors declare no conflict of interest.

### Additional information

No additional information is available for this paper.
